# Editorial: Liquid biopsy biomarkers for cancer detection, treatment monitoring, and clinical outcome prediction

**DOI:** 10.3389/fcell.2026.1970074

**Published:** 2026-09-09

**Authors:** Keerthi Kurma, Zahra Eslami-S, Julie Earl

**Affiliations:** 1 Laboratory of Rare Human Circulating Cells (LCCRH), University Medical Centre of Montpellier, Montpellier, France; 2 CREEC/CANECEV, MIVEGEC (CREES), University of Montpellier, CNRS, IRD, Montpellier, France; 3 European Liquid Biopsy Society (ELBS), Hamburg, Germany; 4 Biomarkers and Personalized Approach to Cancer (BIOPAC) Group, Ramón y Cajal Health Research Institute (IRYCIS), Madrid, Spain

**Keywords:** circulating biomarkers, clinical implementation, liquid biopsy, precision oncology biomarkers, treatment monitoring

## Abstract

**Figure:**
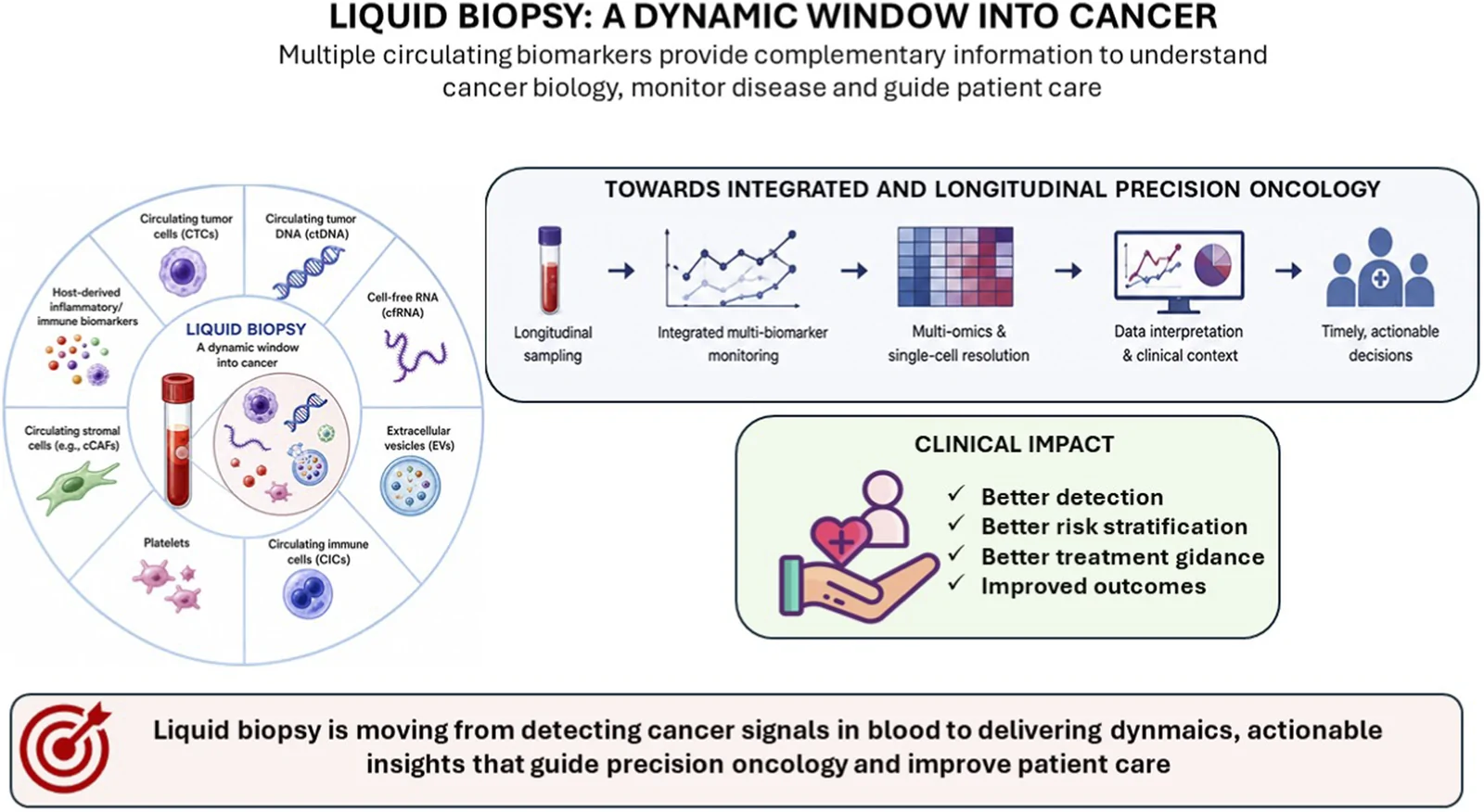

## Introduction

Liquid biopsy has rapidly evolved from a technically innovative approach for detecting tumor derived material in circulation into a broader strategy for understanding cancer as a dynamic and systemic disease. Circulating tumor cells (CTCs), circulating tumor DNA (ctDNA), cell-free RNA (cfRNA), extracellular vesicles (EVs), circulating immune cells (CICs), platlets, circulating stromal cells, and host-derived inflammatory or immune biomarkers can provide complementary information on tumor burden, heterogeneity, treatment response, disease evolution, and clinical outcome.

The central question is therefore no longer simply whether cancer-associated signals can be detected in blood. The challenge is now to determine which signals are clinically meaningful, when they should be measured, how they should be interpreted, and whether they can improve patient management.

## Expanding the biological landscape of circulating biomarkers

CTCs remain among the most informative cellular components of liquid biopsy because they allow direct interrogation of viable tumor-derived cells. However, their rarity, phenotypic plasticity, and methodological complexity continue to limit their broader clinical application. In melanoma, Broseghini et al. highlight both the biological potential of CTC analysis and the need for improved standardization and characterization strategies. Importantly, circulating tumor-derived populations may be substantially more heterogeneous than conventional CTC definitions suggest. In pancreatic cancer, Tagari et al. demonstrate the value of multiparametric phenotyping of CTCs and circulating tumor-associated cells (CTACs), revealing distinct immune-regulatory, signaling, and metabolic features. Such observations support a transition from simple enumeration toward deeper functional and molecular characterization of circulating cells.

The cellular landscape accessible through liquid biopsy also extends beyond malignant cells. Reese Ryterski et al. demonstrate the feasibility of detecting circulating cancer-associated fibroblasts (cCAFs) together with CTCs in melanoma. This emerging concept broadens liquid biopsy from a tumor-cell-centered approach toward interrogation of a wider circulating tumor ecosystem, potentially capturing interactions between malignant and stromal compartments.

In parallel, acellular biomarkers continue to expand the molecular resolution obtainable from blood. ctDNA provides direct access to tumor-associated genomic alterations and is increasingly investigated for molecular profiling, residual disease assessment, treatment monitoring, and detection of resistance. In hepatocellular carcinoma, the systematic review and meta-analysis by Wu et al. further supports the prognostic relevance of ctDNA detection and its potential role in postoperative surveillance and recurrence assessment.

Circulating non-coding RNAs represent another promising biomarker class. In breast cancer, Li et al. examine serum miR-21 alongside VEGF and CA15-3, illustrating the potential of circulating miRNAs to complement established markers for minimally invasive cancer detection.

EVs add another level of biological complexity. These nanoscale carriers transport nucleic acids, proteins, lipids, and signaling molecules between cells and may simultaneously reflect tumor biology and participate in disease progression. The review by Zhao et al. illustrates this dual potential in genitourinary malignancies, positioning EVs both as sources of clinically informative biomarkers and as possible therapeutic platforms.

## Beyond tumor derived signals: capturing the patient tumor ecosystem

An important direction emerging from this Research Topic is the integration of tumor-derived information with systemic characteristics of the host. Cancer progression is shaped by continuous interactions between malignant cells and immune, inflammatory, stromal, metabolic, and vascular compartments. Blood-based markers reflecting systemic inflammation, immune competence, or nutritional status may therefore provide prognostic information that is distinct from, but complementary to, tumor-derived analytes.

This principle is illustrated by Zhang et al., who investigate the C-reactive protein–albumin–lymphocyte index in endometrial cancer and show how readily accessible systemic biomarkers can contribute to individualized prognostic stratification when integrated with clinicopathological variables. Liquid biopsy should therefore not be regarded as a single assay or technology. Rather, it is increasingly becoming a multidimensional framework for capturing both tumor biology and the host response to cancer.

## From analytical performance to clinical utility

Technological progress in liquid biopsy has been remarkable, but analytical sensitivity alone does not establish clinical value. Detecting increasingly rare circulating events or molecular alterations is meaningful only if the information obtained improves diagnosis, prognostic stratification, treatment selection, response assessment, or patient outcome. This distinction is particularly well illustrated by Batra et al., who examine the expanding applications of liquid biopsy across the lung cancer continuum while distinguishing approaches with established clinical relevance from those still requiring prospective validation.

The field must therefore move beyond demonstrating that a biomarker can be measured toward demonstrating when its measurement should alter clinical decision-making.

## Implementation, standardization, and equitable access

Translation into routine oncology also depends on factors extending well beyond biomarker biology. Pre-analytical variability, sample processing, assay sensitivity, threshold definition, data interpretation, and inter-laboratory reproducibility remain critical challenges.

Equally important are cost, reimbursement, institutional resources, clinician familiarity, and access to testing. These Research Topic are highlighted by Li et al., whose real-world assessment of liquid biopsy use in colorectal cancer care demonstrates how financial, infrastructural, and interpretative barriers can influence adoption across healthcare settings. The success of liquid biopsy will therefore depend not only on developing increasingly sensitive assays, but also on ensuring that validated technologies become standardized, interpretable, scalable, affordable, and accessible.

## Toward integrated and longitudinal precision oncology

The next major advances may arise not from identifying a single universal biomarker, but from integrating complementary circulating signals over time. Combining ctDNA, cfRNA, CTCs, CICs, EVs, and host-response biomarkers could provide a multidimensional view of cancer evolution that no individual analyte can achieve alone.

Single-cell technologies, longitudinal sampling, and multi-omic integration will further increase biological resolution. However, increasing analytical complexity must remain aligned with clinical need. The ultimate value of liquid biopsy will not be determined by how much information can be generated, but by whether that information can be transformed into timely, reproducible, and actionable clinical decisions.

Thus, this Research Topic illustrates a field at a pivotal stage of maturation. Liquid biopsy is moving beyond demonstrating that cancer-associated material circulates in blood toward a more ambitious objective: using dynamic circulating information to understand disease evolution, anticipate clinical change, and guide precision oncology.

